# Imaging studies used as aid in the diagnosis of cleidocranial dysplasia. A review

**DOI:** 10.21142/2523-2754-0902-2021-063

**Published:** 2021-06-21

**Authors:** Laura A. Granado-Abasto, Jhoana M. Llaguno-Rubio, Gustavo A. Fiori-Chíncaro, Paola E. Medina-Ocampo

**Affiliations:** 1 Facultad de Odontología, Universidad Mayor de San Simón. Cochabamba, Bolivia. andygranado111@gmail.com Universidad Mayor de San Simón Facultad de Odontología Universidad Mayor de San Simón Cochabamba Bolivia andygranado111@gmail.com; 2 División de Radiología Bucal y Maxilofacial, Carrera de Estomatología, Universidad Científica del Sur. Lima, Perú. academico@ilaeperu.com, gfiori@ilaeperu.com, paomedinaocampo@gmail.com Universidad Científica del Sur División de Radiología Bucal y Maxilofacial Carrera de Estomatología Universidad Científica del Sur Lima Peru academico@ilaeperu.com gfiori@ilaeperu.com paomedinaocampo@gmail.com

**Keywords:** Cleidocranial dysplasia, Cone beam computed tomography, Skeletal dysplasias, Diagnostic imaging, Marie Sainton syndrome, displasia cleidocraneal, tomografía computarizada de haz cónico, displasias esqueléticas, diagnóstico por imagen, síndrome de Marie Sainton

## Abstract

Cleidocranial dysplasia (CCD), also known as Marie-Sainton syndrome, is a rare disorder of autosomal dominant type that presents specific characteristics at the skeletal and dental level. The diagnosis of CCD is based on clinical and radiographic findings. Panoramic, cephalometric and anterior poster radiographs have been used for its diagnosis in dentistry. However, these radiological techniques have limitations, and advances in technology with new imaging studies such as magnetic resonance imaging (MRI) and ultrasound have emerged, contributing to the diagnosis of CCD. Therefore, the aim of this review was to identify and describe current imaging studies that contribute to both the diagnosis and adequate and efficient treatment planning of CCD, and describe the clinical and radiographic characteristics of patients with this syndrome.

## RESUMEN

La displasia cleidocraneal (DCC), también conocida como síndrome de Marie-Sainton, es un trastorno poco común de tipo autosómico dominante, que presenta características específicas a nivel esquelético y dental. El diagnóstico de DCC se basa en hallazgos clínicos y radiográficos. Las radiografías panorámicas, cefalométricas y posteroanteriores se han utilizado para su diagnóstico en el área de la odontología, pero con los avances de la tecnología y debido a las limitaciones de estas técnicas radiológicas han surgido nuevos estudios de imagen como la resonancia magnética (RM) y la ecografía, que contribuyen al diagnóstico de DCC. Por lo tanto, el propósito de esta revisión fue identificar y describir los estudios de imagen actuales que aportan tanto al diagnóstico como a la planificación del tratamiento adecuado y eficiente de la DCC, y permiten describir las características clínicas y radiográficas de los pacientes con este síndrome.

## INTRODUCTION

Cleidocranial dysplasia (CCD) also known as Marie-Sainton syndrome, is an autosomal dominant disorder characterized by facial, dental and skeletal malformations ^1^^-^^3^. The term CCD derives from the Greek words: cleido (clavicle), cranial (head) and dysplasia (abnormal formation) ^2^. The incidence of this syndrome is 1 in 1,000,000 live newborns and has no preference for race or sex ^4^. The main clinical characteristics of patients with CCD are hypoplastic or aplastic clavicles, delayed closure of fontanelles, and the presence of multiple supernumerary teeth ^2^^,^^5^^-^^7^.

Early diagnosis of CCD allows adequate treatment planning to improve the quality of life of the patients ^8^^,^^9^. Previously, the imaging studies available for the diagnosis of CCD in dentistry were based on panoramic, cephalometric and posteroanterior radiographs. Unfortunately, these studies only provide a general vision and, in many cases, are distorted due to factors such as image magnification, superposition and patient position and are also insufficient to evaluate the entire craniofacial massif ^10^^,^^11^.

Cone beam computed tomography (CBCT) is a useful auxiliary tool for the diagnosis of CCD and to direct treatment planning ^(11, 12)^. Other useful studies for the diagnosis of CCD are magnetic resonance imaging (MRI) and ultrasound. It is important to carry out differential diagnosis of CCD with other entities such as Crane-Heise syndrome, mandibuloacral dysplasia, pycnodysostosis, Yunis-Varon syndrome, and hypophosphatasia, which share similar clinical and radiographic characteristics ^13^^-^^15^. Management of orofacial manifestations requires the intervention of a multidisciplinary team made up of specialists in orthodontics, oral rehabilitation, radiology and maxillofacial surgery ^1^^,^^11^^,^^1^.

There are reports on the use of CBCT, MRI and ultrasound in other diseases. However, to our knowledge no study has described their use in the diagnosis of CCD in dentistry. Therefore, the objective of this review was to provide an updated description of the imaging studies available that contribute to the diagnosis of CCD as well as describe the clinical and radiographic characteristics of this syndrome.

## MATERIAL AND METHODS 

A literature search to update important concepts of CCD was conducted in international scientific journals using Medline via Pubmed, Scopus, Scielo and LILACS, until June 20, 2020 using the following keywords: Cleidocranial dysplasia, Cone beam computed tomography, Skeletal dysplasias, Diagnostic imaging, Marie Sainton syndrome. Original articles, case reports and literature reviews were included and letters to the editor were excluded. 

### Clinical and 2D Radiographic Characteristics of Cleidocranial Dysplasia

This syndrome is caused by a mutation in the *RUNX2* gene, which is responsible for osteoblastic differentiation, chondrocyte maturation, and proper bone formation ^6^^,^^8^. It mainly affects the bones that are formed by intramenbranous ossification, such as the skull and clavicles, with these patients presenting a characteristic facial appearance ^8^. The general clinical characteristics of patients with CCD are the presence of hypoplastic or aplastic clavicles, which allows these patients to rotate the shoulders towards the midline, hypertelorism, a narrow bell-shaped thorax, flat feet, genu valgum, problems in the spine such as scoliosis and a wide symphysis pubis ^7^. These individuals are generally short in stature, being 7.5 to 15 cm shorter than their unaffected siblings, and can present recurrent respiratory tract infections and hearing loss, while their general health and intellect are not affected ^8^^,^^13^.

The most relevant craniofacial characteristics are a delay in the closure of the cranial sutures. In particular, these patients present permeability of the anterior fontanelle, which can remain open until adulthood ^4^, and the hypoplastic maxillary bone has a tendency to present cleft or ogival palate. Individuals with CCD have a flat wide nasal bridge as well as protuberance of the frontal, parietal and occipital bones. These patients also have a tendency to being brachycephalic and present delayed ossification of the skull and lack of development of the paranasal sinuses ^8^.

Manifestations at the dental level include the delay or absence of exfoliation of the deciduous teeth, enamel hypoplasia, late eruption of permanent teeth or retention of the same, with alteration in their shape and number. They commonly present multiple supernumerary teeth which do not erupt due to lack of space or failure in the bone resorption process, which leads to tooth crowding, malocclusion and even the development of dentigerous cysts due to retention of the teeth ^1^^,^^7^^,^^15^.

**Figure 1:**
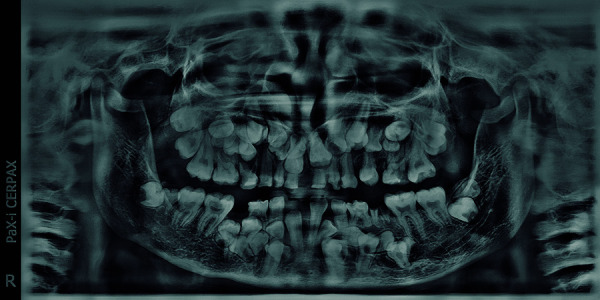
Panoramic radiograph showing multiple supernumerary teeth and parallel ascending branches.

Among the imaging studies used for the diagnosis of CCD, panoramic, cephalometric and posteroanterior radiographs have been the most frequently used, since they allow two of the main characteristics of this syndrome to be observed; the presence of several supernumerary teeth and the lack of closure of the sutures and cranial fontanelles. It is worth mentioning that chest X-rays are also very useful in the diagnosis since they show the absence or either uni or bilateral hypoplasticity of the clavicles ^12^. Panoramic radiography is useful for examining the general condition of the teeth, including tooth germs, bone resorption, and for evaluating temporomandibular joint disorders and even certain cysts and tumors ^16^. The distinctive radiographic characteristics of CCD seen in panoramic radiographs include the presence of multiple supernumerary teeth (Figure 1), shape anomaly of the ascending mandibular ramus where the anterior and posterior border are parallel, and a thick bone trabeculate is also usually observed with increased bone density and sclerosis of the alveolar bone, as well as a U-shaped sigmoid notch. The coronoid process can be very thin, and the zygomatic arch and bone are very thin and sometimes discontinuous. The maxillary sinuses are usually hypoplastic with features of pneumatization and may even be absent ^6^^,^^15^. It is common to observe a hypoplastic maxillary bone, and in some cases, there is a narrow distance between the floor of the orbit and the crowns of the erupting teeth, which can confound the orbit with the maxillary sinus ^8^.

**Figure 2:**
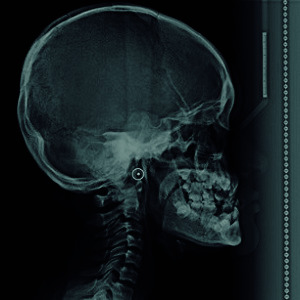
Lateral skull radiograph showing mandibular prognathism, multiple supernumerary teeth, and increased density and thickness of the contour of the cranial vault.

On the other hand, cephalometric or lateral skull radiography is indicated to evaluate facial growth, paranasal sinuses, hard palate and trauma. The radiographic characteristics of CCD that can be observed in this type of radiography are hypoplasia of the maxillary bone with multiple retained and supernumerary teeth, lack of development of the mastoid cells, and prominence of the frontal and occipital bone, in addition to the type of occlusion and profile of these patients ^17^ (Figure 2).

Posteroanterior radiographs show the open cranial sutures and fontanelles, as well as the lack of development of the paranasal sinuses, and the presence of multiple worminan bones. The latter are formed from ossification centers and can vary in shape, number, size and location, although they are commonly located at the level of the sutures and cranial fontanelles ^6^^,^^12^^,^^17^. 

### Current Imaging Studies for the diagnosis of Cleidocraneal Dysplasia and the Radiographic Characteristics

**Figure 3:**
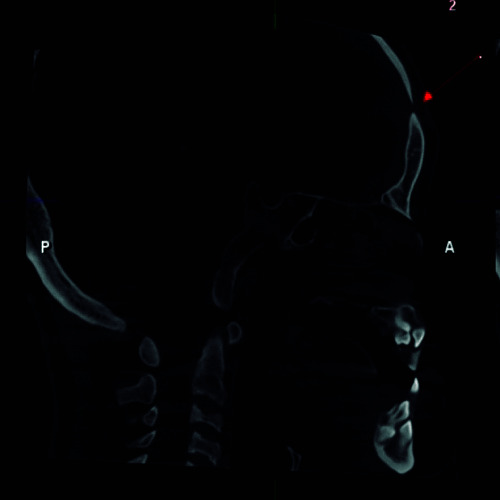
Sagittal section of CBCT of a patient with CCD showing the lack of closure of the frontal suture.

**Figure 4:**
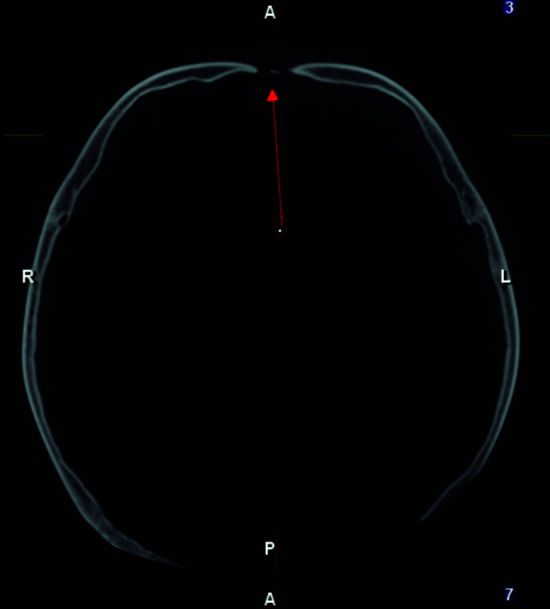
Axial CBCT section of a patient with CCD showing the open frontal suture.

**Figure 5:**
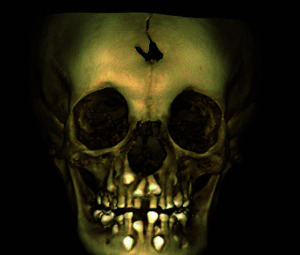
3D reconstruction of a patient with CCD showing the open frontal suture.

To overcome the limitations presented by two-dimensional (2D) radiographs, new imaging methods have emerged such as cone beam computed tomography (CBCT), which offers a high resolution of the image in three dimensions of space ^12^ (Figures 3,4,5). In 1998 Mozzo and collaborators introduced CBCT in the field of dentistry and maxillofacial radiology seeking to reduce the cost and radiation dose to which patients were exposed with traditional medical computed tomography ^18^. The use of CBCT in patients with CCD allows the evaluation of supernumerary and retained teeth in relation to their shape, size, number, position and relationship with adjacent anatomical structures ^33^. It also allows examination of the facial morphology, the maxillary sinuses which are usually small or underdeveloped, and measurement of the vertical and horizontal dimensions of the middle third of the face, which are observed as being reduced in patients with CCD, leading to medium facial hypoplasticity ^19^. It is useful to observe the degree of bone and root resorption of the teeth ^2^^,^^11^. Likewise, CBCT provides tomographic sections of the hypoplasticity of the maxillary bone, the presence of Wormian bones, the zygomatic arch displaced downwards, the hypoplasticity of the odontoid process, and on rare occasions, fusion of the stapes and the Eustachian tube is also observed ^6^^,^^11^. According to what has been described, the formation of dentigerous cysts is possible due to the retention of teeth. CBCT allows evaluation of these lesions in extension and proximity with adjacent structures, since they can displace or reabsorb neighboring teeth, and in certain cases they may show odontomas, which are associated with retained deciduous teeth and impacted permanent teeth ^20^^,^^13^^,^^30^.

Regarding MRI in dentistry, its use in the diagnosis of CCD has not been sufficiently studied but it is not a completely new topic ^21^, with several reports on its clinical application in the diagnosis of this syndrome being available in the literature ^22^. In the prenatal diagnosis of bone dysplasias such as CCD, MRI has had very limited use, despite being indicated to evaluate skeletal abnormalities at between 18 and 22 weeks of gestation ^34^. MRI can evaluate the alterations in the temporomandibular joint that these patients may present as well as determine the exact location of the mandibular nerve. It can even allow the evaluation of the pulp and periapical tissue of the teeth as well as the presence of caries, dental structures and micrognathia. On the other hand, brain MRI of these patients shows brachycephalia and prominence of the frontal, occipital and parietal bones ^1^^,^^21^.

It has been reported in the literature that MRI is useful for the prenatal diagnosis of cleft palate, an anomaly which usually affects patients with CCD. It also allows the nasopharynx and oropharynx to be evaluated, providing valuable data to foresee and establish adequate treatment even before birth ^2^^,^^3^.

Another useful study in the diagnosis of CCD during gestation is ultrasound, which has been used as a diagnostic imaging method since 1950. Ultrasound is useful for identifying skeletal and orofacial anomalies of the fetus during pregnancy, and according to the study by Salazar *et al*., 50% of fetal anomalies are detected during gestation by ultrasound. Ultrasound presents a high sensitivity and specificity for anomalies such as cleft palate, palatal fissures, micrognathia and malformations of the nasal bones, which are manifestations of CCD ^25^. The ultrasound findings observed in patients with skeletal dysplasias such as CDC include rhizomelia, small chest, frontal prominence, widening of the ribs, facial dysmorphisms, acromelia, brachydactyly, brachycephaly, absent nasal bones, and flat wide nasal bridge in addition to hypomineralization of the skull and spine ^24^^,^^33^. 

Studies in which the prenatal diagnosis of CCD was made by ultrasound at week 14 of gestation describe clavicular hypoplasty, hypomineralization of cranial bones and pelvis, as well as wide cranial sutures and the absence of the squamous portion of the temporal bone ^31^.

Another alternative for the prenatal diagnosis of bone dysplasia is low-dose fetal computed tomography, which allows evaluating bone structures with high precision from the second trimester of pregnancy. Although this technique is new and few cases have been reported, its application has shown very good sensitivity to detect and evaluate intrauterine bone abnormalities. Indeed, the sensitivity of this technique is greater than that of ultrasound, ranging between 40% and 60% ^32^. It should be noted that the definitive diagnosis of CCD is made based on the clinical and radiographic findings observed after birth or by molecular studies ^1^.

### Advantages and Disadvantages of the Current vs. Conventional 2D images for the Diagnosis of Cleidocranial Dysplasia

2D radiographs are the imaging studies most commonly used in the daily performance of dentistry, being the first choice as an auxiliary diagnostic tool, with a cost that is much lower than that of CBCT and MRI. These are simple radiographic methods, which produce low exposure to radiation, serve as a legal document as they cannot be subject to modifications by software as in the case of conventional radiographs ^26^. They require correct and precise patient positioning as well as careful processing and handling of radiographic films ^16^. The disadvantages of 2D radiographs are image distortion and magnification and the superpositioning of anatomical structures, and it should be taken into account that they project a 2D image of 3D structures ^11^^,^^15^.

CBCT was introduced as a new diagnostic method in the area of dentistry. This technique uses a cone-shaped X-ray beam and has the ability to reconstruct images free of distortion and magnification in three dimensions of space ^18^. Among its advantages is the ability to select the field of view, which reduces unnecessary irradiation to the patient, offers high image precision, allows accurate observation of the structures of the craniofacial massif, and the acquisition time. The duration of image acquisition varies from approximately 5 to 40 seconds, which is comparable to that of panoramic digital radiography. It also allows the images obtained to be processed on the viewer and accuracy measurements can be made using the software ^18^. The advantages of CBCT over traditional clinical computed tomography are that the image quality is much greater, it has a lower cost and the radiation dose is relatively lower ^27^.

On the other hand, in comparison with X-rays, one of the disadvantages of CBCT is the radiation dose. X-rays use electromagnetic radiation with high penetrating power, which can cause damage to human body tissues. The radiation dose to which a patient is exposed during the acqusitiion of CBCT is measured in sieverts (Sv), and is of approximately 48 to 1073 µSv in CBCT, which is higher compared to that of a panoramic radiograph (2.7 to 24.3 µS) and lateral skull radiograph (approximately 6 µS) ^28^. The radiation dose of CBCT is 16 times greater than that of a panoramic radiograph, being 30 times greater in children than in an average adult, thereby making it important to evaluate the risk-benefit factor according to patient age and make rational use of this imaging resource in a justified and responsible manner ^26^. Another disadvantage of CBCT is the presence of noise or artifacts caused by metallic structures such as implants, braces, crowns and intra-radicular posts, which reduces the clarity as well as the quality and resolution of the images obtained ^29^.

In recent years, the use of MRI as a diagnostic tool has been introduced in the field of dentistry. It is a non-invasive technique that is used to evaluate different diseases and soft and hard tissue injuries. It allows obtaining real time images in different spatial planes and has excellent soft tissue contrast capabilities ^22^. Among its advantages is the ability to reconstruct images with high tissue enhancement, allowing tissue to be observed at different angles and planes without the need to change the position of the patient, and, moreover, it does not produce ionizing radiation. The biggest disadvantage, however, is the high cost and the time that the patient must remain still during its application, which is of approximately 3 minutes, being uncomfortable and tiring ^21^^,^^23^.

## CONCLUSIONS

Patients with CCD present a triad of clinical characteristics, two of which are clearly evident, and these are the presence of supernumerary teeth, and delayed closure of sutures and cranial fontanelles. The presence of hypoplastic clavicles is also shown in chest X-ray which continues to be useful for the diagnosis of this syndrome. Nonetheless, technological advances have led to the use of CBCT, MRI and ultrasound, allowing the description of new imaging characteristics which had not previously been described with the use of 2D radiographies in patients with CCD. 

Although these new imaging studies have some disadvantages such as cost and radiation dose compared to 2D radiographs, 3D evaluation provides the advantage of avoiding image superimposition and not only contributes to the diagnosis of CCD but also helps to orient treatment planning. In addition, MRI and ultrasound are useful for the prenatal diagnosis of CCD.
